# *Yersinia pseudotuberculosis* Septicemia and HIV

**DOI:** 10.3201/eid1107.041268

**Published:** 2005-07

**Authors:** Maria Grazia Paglia, Silvia D'Arezzo, Anna Festa, Cosmo Del Borgo, Laura Loiacono, Andrea Antinori, Giorgio Antonucci, Paolo Visca

**Affiliations:** * National Institute for Infectious Diseases "Lazzaro Spallanzani," Rome, Italy;; † University "Roma Tre," Rome, Italy

**Keywords:** Opportunistic infection, HIV, Yersinia pseudotuberculosis, Molcular epidemiology, Immunosuppression

## Abstract

Two cases of community-acquired septicemia caused by serotype-O1 *Yersinia pseudotuberculosis* were diagnosed in middle-aged, HIV-positive, immunodeficient patients during an 8-month period. Bacterial isolates were genetically indistinguishable, but no epidemiologic link between the 2 patients was established. HIV-related immunosuppression should be regarded as a risk factor for *Y*. *pseudotuberculosis* septicemia.

*Yersinia pseudotuberculosis* is a rare cause of disease in humans. Animals, food, and the abiotic environment are *Y*. *pseudotuberculosis* reservoirs from which epizootic and human infection may arise (1). A geographic gradient of *Y*. *pseudotuberculosis* isolation rates has been reported in Europe (1,2), with a 0.05% recovery rate from stools of patients with acute enteritis in Italy (3). The organism can also cause mesenteric lymphadenitis, which mimics appendicitis, or infection at other body sites that occasionally leads to postinfectious sequelae such as reactive arthritis and erythema nodosum (1). About 60 cases of *Y*. *pseudotuberculosis* septicemia have been reported thus far, mainly in patients with underlying conditions such as hepatic cirrhosis, malignancy, diabetes, aplastic anemia, thalassemia, and iron overload (1,4,5). We recently reported the first case of *Y*. *pseudotuberculosis* septicemia in a severely immunocompromised, HIV-positive patient (6). Here, a second case of *Y. pseudotuberculosis* septicemia in an HIV-infected outpatient attending the same hospital is described. The unique molecular type of both *Y*. *pseudotuberculosis* isolates and the atypical clinical course of infection will be comparatively discussed.

## The Study

The first case has recently been reported (6) and is briefly reviewed here. Patient 1 was a 42-year-old woman with HIV infection since 1987 (Centers for Disease Control and Prevention [CDC] Classification C3). In June 2003, she was admitted to the National Institute for Infectious Diseases, Rome, from prison because of high fever and confusion. Physical examination showed a temperature of 39.5°C, abnormal mental status, and oral candidiasis, but no gastrointestinal symptoms. Her history included lack of response to highly active antiretroviral therapy (HAART), and she exhibited HIV viremia of 413,624 copies/mL, a low CD4+ cell count (5/mm^3^), and leukopenia (3.0 × 10^3^/mm^3^). Laboratory values were altered for aminotransferases (aspartate aminotransferase, 273 U/L; alanine aminotransferase, 77 U/L), hemoglobin (7.0 g/dL), erythrocyte sedimentation rate (136 mm in the first hour), and platelet count (34 × 10^3^/mm^3^). Multiple blood cultures yielded growth of *Y*. *pseudotuberculosis*. Stool cultures and test results for antibodies against *Y*. *pseudotuberculosis* were negative. Intravenous ceftriaxone therapy was started at admission, with total remission of symptoms in 4 days. No recurrence of *Y*. *pseudotuberculosis* infection was observed during a 1-year follow-up period.

Patient 2 was a 54-year-old man who was admitted to the same hospital in February 2004 because of sudden fever and confusion. He tested HIV-positive in 1993 (CDC Classification B3), and his CD4+ cell count nadir was 121 cells/mm^3^. His recent antiretroviral therapy was a combination of stavudine, lamivudine, and nelfinavir. HCV-related liver cirrhosis was diagnosed 3 years before this admission. His condition was routinely followed up in the outpatient unit, and he had been hospitalized 1 month earlier for culture-negative pneumonia in the left lower lobe. On admission, the patient had a high fever (41°C), chills, and abnormal mental status with somnolence. Results of a chest radiograph, nuclear magnetic resonance imaging of the brain, and echocardiogram were normal. HIV viremia level was <50 copies/mL and CD4+ cell count was 204 cells/mm^3^. Laboratory values were notable for leukocytosis (leukocytes, 14 × 10^3^/mm^3^), anemia (hemoglobin, 10 g/dL), and thrombocytopenia (platelets, 69 × 10^3^/mm^3^). Levels of C reactive protein, aminotransferases, blood glucose, creatinine, urea nitrogen, and electrolytes were within the normal range. Liver cirrhosis was classified as Child-Pugh group B, score 7. Urine was negative for opioids and cocaine metabolites. On second day postadmission, multiple blood cultures became positive for *Y*. *pseudotuberculosis*, while stool cultures were negative. Intravenous ceftriaxone therapy was begun at admission, with total remission of symptoms within 3 days. He was discharged on day 10 and continued intravenous ceftriaxone therapy for 4 weeks (2 g daily) as an outpatient. At the final clinical observation, 6 months later, the patient was free of clinical symptoms and had no recurrence of *Y*. *pseudotuberculosis* infection.

Blood culture isolates from both patients were identified with >99% confidence as *Y*. *pseudotuberculosis* by using both the API20E-Rapid test (bioMérieux, Marcy l'Etoile, France) and the BD Phoenix system (Becton Dickinson, Sparks MD, USA). Isolates showed an extended spectrum of antimicrobial susceptibility (sensitive to β-lactams, monobactams, aminoglycosides, quinolones, and sulfonamides). O-serotyping with commercial antisera (Denka Seiken, Tokyo, Japan) identified both organisms as *Y*. *pseudotuberculosis* serotype O1, consistent with the high frequency of this serotype among strains isolated from patients with septicemia in Europe (2).

The 16S ribosomal DNA (rDNA) sequences were identical in the 2 isolates and optimally matched (>99% identity) the 16S rDNA of the *Y*. *pseudotuberculosis* type strain ATCC 29833 (GenBank accession no. AF366375). To differentiate the strains at the genome level, amplified fragment length polymorphism and arbitrarily primed polymerase chain reaction (PCR) were performed (7–9). While these methods are highly discriminatory and detect DNA polymorphisms to the strain level, no differences between the 2 clinical isolates were observed (Figure, panels A and B).

**Figure:**
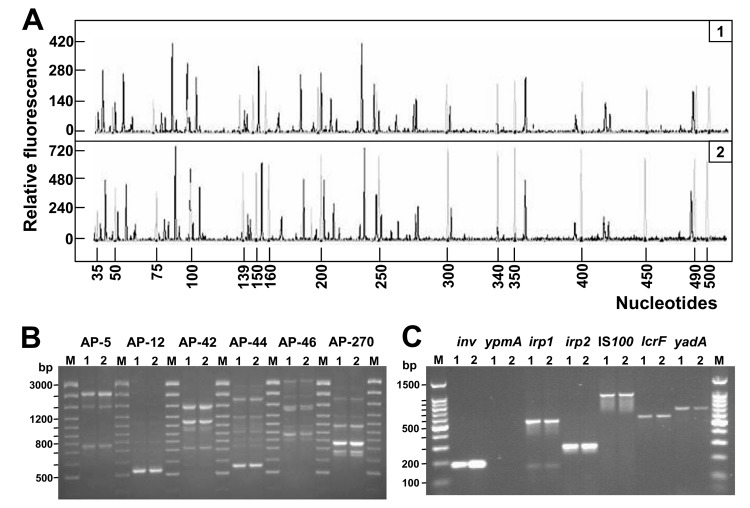
Genetic fingerprinting and detection of virulence genes of *Yersinia pseudotuberculosis* isolates. A) Fluorescent amplified fragment length polymorphism (AFLP) analysis of *Y*. *pseudotuberculosis* DNA (black electropherogram; 1 and 2 refer to patient number). Reactions were performed as indicated in the AFLP Microbial Fingerprinting kit (Applied Biosystems, Foster City, CA, USA). Reference DNA from *Escherichia coli* W3110 (Applied Biosystems) was used as internal control (gray electropherogram). Separation and detection of the AFLP fragments were performed with the Applied Biosystems model 3100 capillary electrophoresis system equipped with a 36-cm capillary loaded with the POP-4 polymer. Size determinations of the labeled DNA fragments were performed automatically with the Genescan Analysis 3.0 software (Applied Biosystems). B) Arbitrarily primed polymerase chain reaction (AP-PCR) analysis with a set of 6 oligonucleotides: AP5 (5´-TCCCGCTGCG-3´), AP12 (5´-CGGCCCCTGC-3´), AP42 (5´-AACGCGCAAC-3´), AP44 (5´-AGCCAGTTTC-3´), AP46 (5´-GAGGACAAAG-3´), and AP270 (5´-TGCGCGCGGG-3´) (8,9). Amplification patterns of DNA from the 2 clinical isolates are shown: lane 1, patient 1; lane 2, patient 2. M, molecular weight marker. The numbers on the left indicate the length (in base pairs) of the reference ladder. Primers are indicated on top. C) Detection of *Y*. *pseudotuberculosis* virulence genes. Primers and PCR conditions have been described elsewhere (2,6). Lane 1, patient 1; lane 2, patient 2. Lane M, molecular weight marker. The numbers on the left indicate the length (in base pairs) of the reference ladder. Target genes are indicated on top.

To gain insight into the pathogenic potential of the 2 isolates, we assessed the presence of pYV (70-kb virulence plasmid), high pathogenicity island (HPI), and *Y*. *pseudotuberculosis*–derived mitogen (YPM) genetic markers by PCR (2,6). Both isolates harbored a characteristic repertoire of virulence determinants. Correctly sized PCR products were obtained for *inv* (chromosome-borne), *irp1*, *irp2*, IS*100* (HPI-borne), *lcrF*, and *yadA* (plasmidborne) genes (Figure, panel C). The identity of PCR products was confirmed by direct DNA sequencing. Interestingly, both isolates were negative for the *ypmA* (chromosome-borne) gene, consistent with the genetic instability of this marker which is typically absent in strains from Western countries (2,10). Reducing PCR stringency for *ypmA* yielded a 237-bp amplicon, which resulted from mispriming of oligonucleotides on the *Y*. *pseudotuberculosis hmp* flavohemoprotein gene (data not shown).

Molecular typing and virulence gene probing indicate that both cases resulted from indistinguishable *Y*. *pseudotuberculosis* strains, which raises the suspicion of a common source of infection. However, no other cases of *Y*. *pseudotuberculosis* infection were diagnosed in our institute or reported to the National Center for Enteropathogenic Bacteria, Istituto Superiore di Sanità, Rome, from January 2003 to June 2004 (I. Luzzi, pers. comm.). In September 2004, patients were interviewed about their lifestyle, illness, food and fluid consumption, or animal exposure in the 2 weeks preceding hospitalization. Nosocomial infection and direct contact between patients were ruled out, which suggested that infection had been independently acquired in the community. Both patients denied any contact with wild or domestic animals. Patient 1 had not been released from detention in the 6 months preceding hospitalization and regularly consumed collective meals in prison. However, prison infirmary records did not show an increased frequency of abdominal symptoms or fever among ≈500 inmates from May to July 2003. Patient 2 was a heavy smoker who had been abusing alcohol and illicit drugs until 2001. He lived alone and had a seafood meal 4 days before admission. Thus, although infection may have been acquired from contaminated food or fluid, questions regarding the actual source of bacteria and the extent of exposure remain unanswered.

## Conclusions

AIDS is a known risk factor for *Y*. *enterocolitica* infection (11), but no link between HIV and *Y*. *pseudotuberculosis* infection has yet been proposed. Yersiniae have an impaired iron metabolism, and for this reason, they rarely cause sepsis in patients without iron overload, which is often secondary to alcoholism, asplenia, hemochromatosis, thalassemia major, or tobacco smoking (1). In our 2 patients, no clinical evidence indicated iron overload, and a diagnosis of hemochromatosis was excluded. The most important risk factor was HIV-related severe immunodeficiency. Patient 1 was severely immunocompromised despite HAART, while patient 2 responded poorly to HAART and had hepatitis C–related liver cirrhosis.

Although the clinical management of *Y. pseudotuberculosis* septicemia is often difficult and mortality rates are high (≈75%), despite antimicrobial drug therapy (1), both our patients responded unexpectedly well to ceftriaxone therapy and promptly recovered. During infection, *Y*. *pseudotuberculosis* directly manipulates lymphocyte signaling and activation by expressing different virulence factors (12). The pYV plasmid-encoded Yop proteins have been implicated in lymphocyte suppression through down-regulation of co-stimulatory molecules (13) and impairment of nitric oxide, tumor necrosis factor (TNF)-α, and proinflammatory cytokine production (14). Conversely, YPM superantigen(s) contribute to systemic illness by activating a large proportion of T cells (essentially CD4+) and inducing proinflammatory cytokines such as TNF-α, TNF-β, γ-interferon, and interleukins 1 and 6, as in toxic shock syndrome (15). Our experimental data show that the 2 clinical strains were positive for all the virulence genes tested, except for *ypm*A. Even considering the different degree of immunodeficiency between the 2 patients, we speculate that impairment of immune response secondary to HIV infection may have increased the susceptibility to *Y*. *pseudotuberculosis* infection while mitigating the septic shock sequelae. Accordingly, the inflammatory response consequent to T-cell activation may have been attenuated by the deficiency of CD4+ cells in both patients, concomitant with the lack of YPM expression by both *Y*. *pseudotuberculosis* isolates. In conclusion, these 2 cases indicate that *Y*. *pseudotuberculosis* is an emerging pathogen in HIV patients and remind us that septicemia in these patients can exist without prodromic symptoms. They also alert us to the local circulation of a pathogenic *Y*. *pseudotuberculosis* strain whose natural reservoir remains so far unknown.
